# Overexpression of miR-9 in mast cells is associated with invasive behavior and spontaneous metastasis

**DOI:** 10.1186/1471-2407-14-84

**Published:** 2014-02-11

**Authors:** Joelle M Fenger, Misty D Bear, Stefano Volinia, Tzu-Yin Lin, Bonnie K Harrington, Cheryl A London, William C Kisseberth

**Affiliations:** 1 Department of Veterinary Clinical Sciences, Columbus, USA; 2 Department of Veterinary Biosciences, Columbus, USA; 3 Department of Molecular Virology, Immunology, and Medical Genetics, The Ohio State University, Columbus, OH, USA; 4 Division of Hematology and Oncology, Department of Internal Medicine, University of California-Davis, Sacramento, CA, USA

**Keywords:** Mast cell, microRNA, miR-9

## Abstract

**Background:**

While microRNA (miRNA) expression is known to be altered in a variety of human malignancies contributing to cancer development and progression, the potential role of miRNA dysregulation in malignant mast cell disease has not been previously explored. The purpose of this study was to investigate the potential contribution of miRNA dysregulation to the biology of canine mast cell tumors (MCTs), a well-established spontaneous model of malignant mast cell disease.

**Methods:**

We evaluated the miRNA expression profiles from biologically low-grade and biologically high-grade primary canine MCTs using real-time PCR-based TaqMan Low Density miRNA Arrays and performed real-time PCR to evaluate miR-9 expression in primary canine MCTs, malignant mast cell lines, and normal bone marrow-derived mast cells (BMMCs). Mouse mast cell lines and BMMCs were transduced with empty or pre-miR-9 expressing lentiviral constructs and cell proliferation, caspase 3/7 activity, and invasion were assessed. Transcriptional profiling of cells overexpressing miR-9 was performed using Affymetrix GeneChip Mouse Gene 2.0 ST arrays and real-time PCR was performed to validate changes in mRNA expression.

**Results:**

Our data demonstrate that unique miRNA expression profiles correlate with the biological behavior of primary canine MCTs and that miR-9 expression is increased in biologically high grade canine MCTs and malignant cell lines compared to biologically low grade tumors and normal canine BMMCs. In transformed mouse malignant mast cell lines expressing either wild-type (C57) or activating (P815) KIT mutations and mouse BMMCs, miR-9 overexpression significantly enhanced invasion but had no effect on cell proliferation or apoptosis. Transcriptional profiling of normal mouse BMMCs and P815 cells possessing enforced miR-9 expression demonstrated dysregulation of several genes, including upregulation of CMA1, a protease involved in activation of matrix metalloproteases and extracellular matrix remodeling.

**Conclusions:**

Our findings demonstrate that unique miRNA expression profiles correlate with the biological behavior of canine MCTs. Furthermore, dysregulation of miR-9 is associated with MCT metastasis potentially through the induction of an invasive phenotype, identifying a potentially novel pathway for therapeutic intervention.

## Background

Mast cell-associated malignancies are important diseases in both humans and dogs [1,2] and are characterized by activating mutations in KIT in both species. More than 90% of human patients with systemic mastocytosis carry the D816V mutation in *KIT*[3] which results in constitutive activation of KIT signaling and plays a major role in the proliferative phenotype. A functionally identical mutation (D814V) is found in transformed mast cell lines from rodents [4,5]. Similarly, approximately 30% of dogs with high-grade cutaneous mast cell tumors (MCTs) possess activating internal tandem duplications (ITDs) in the KIT juxtamembrane (JM) domain [6,7]. More recently, activating mutations in the extracellular domain of KIT (exons 8 and 9) have also been identified in a proportion of canine MCTs [8]. While the role of KIT dysfunction in mast cell neoplasia has been well described, little is known regarding additional molecular mechanisms that may contribute to invasion and metastasis of malignant mast cells.

The expression of matrix metalloproteinases (MMPs), a family of enzymes involved in the degradation and remodeling of extracellular matrix, has been implicated in the neoplastic transformation of mast cells. Normal canine bone marrow-derived mast cells (BMMCs) produce large quantities of inactive and active MMP9 in response to various stimuli while releasing little detectable MMP2 [9]. Neoplastic mast cells are known to produce both MMP2 and MMP9 [10] suggesting that the ability to produce MMP2 may be a feature acquired by malignant mast cells. Furthermore, high-grade MCTs express significantly higher levels of MMP9 in proactive and active forms, which has been proposed to be associated with the high degree of malignant behavior of these tumors [10,11]. More recently, characterization of the proteome of primary canine low-grade MCTs and aggressive, high-grade MCTs identified differentially expressed proteins between the two groups [12]. Several stress response proteins (HSPA9, TCP1A, TCP1E) and cytoskeletal proteins associated with actin remodeling and cell migration (WDR1) were significantly up-regulated in high-grade MCTs.

MicroRNAs (miRNAs) are highly conserved, noncoding RNAs that serve as important regulators of gene expression. It is well established that miRNA expression is altered in many human malignancies and that miRNAs function as tumor suppressor genes or oncogenes through dysregulation of target genes [13]. Currently there is limited information regarding the potential role of miRNA dysregulation in malignant mast cell disease. Several miRNAs appear to play an important role in normal murine mast cell differentiation [14] and following activation of murine mast cells, up-regulation of the miR-221-222 family influences cell-cycle checkpoints, in part by targeting p27^Kip1^[15]. Basal levels of miR-221 contribute to the regulation of the cell cycle in resting mast cells. However, its effects are activation-dependent and in response to mast cell stimulation; miR-221 regulates degranulation, cytokine production, and cell adherence [16]. More recent studies have demonstrated roles for miR-539 and miR-381 in mediating a novel regulatory pathway between KIT and microphthalmia-associated transcription factor in normal and malignant mast cells [17].

The purpose of this study was to investigate the potential role of miRNA dysregulation in the biologic behavior of primary canine MCTs. We found that unique miRNA expression profiles correlate with the biological behavior of primary canine MCTs and that miR-9 was significantly overexpressed in aggressive MCTs compared to benign MCTs. Furthermore, enforced miR-9 expression in murine mastocytoma cell lines and normal murine BMMCs with low basal levels of miR-9 enhanced invasion and induced the expression of several target genes associated with metastasis, including chymase (CMA1) and heparinase (HSPE). These data suggest that miR-9 overexpression may contribute to the invasive phenotype of malignant mast cells thereby providing a potentially novel pathway for therapeutic intervention in malignant mast cell disease.

## Methods

### Cell lines, primary cell cultures, primary tumor samples

Mouse P815 (D814V *KIT* mutation) and C57 (wild-type *KIT*) cell lines were provided by Dr. Stephen Galli (Stanford University). The canine BR (activating point mutation L575P in the JM domain of *KIT*) and C2 (*KIT* ITD mutation in the JM domain) cell lines were provided by Dr. Warren Gold (Cardiovascular Research Institute, University of California- San Francisco). Cell lines were maintained in RPMI 1640 (Gibco® Life Technologies, Grand Island, NY, USA) supplemented with 10% fetal bovine serum (Gibco® Life Technologies) and antibiotics (Gibco® Life Technologies). Mouse BMMCs were generated from bone marrow from C57/B6 wild-type mice as previously described [9]. Canine BMMCs were generated from 2 dogs and maintained in Stemline (Sigma-Aldrich, St. Louis, MO, USA) medium supplemented with recombinant canine stem cell factor (R & D Systems, Minneapolis, MN, USA) as previously described [18]. Protocols for collection of murine bone marrow and canine bone marrow were approved by the Ohio State University (OSU) Institutional Care and Use Committee (IACUC), protocols 2009A0204 and 2010A0015, respectively. Canine MCTs were obtained from 24 different affected dogs presented to the OSU Veterinary Medical Center and University of California-Davis (UCD) Veterinary Teaching Hospital. Tumor sample collections were performed in accordance with established hospital protocols and approved by respective IACUC at both OSU and UCD. Clinical outcome data, including sex, breed, primary tumor location, recurrence and metastasis, histopathologic grade, mitotic index, and outcome was available for all dogs (see Additional file 1). Tumors obtained from dogs that were adequately controlled with surgery alone and did not develop or die from metastatic mast cell disease were considered biologically low-grade tumors (benign). Tumors from dogs that developed aggressive, metastatic mast cell disease which resulted in their death were classified as biologically high-grade tumors.

### Quantitative reverse-transcription-PCR profiling of mature miRNA expression in MCT biopsies

Total RNA was isolated by the Trizol method (Invitrogen, Carlsbad, CA, USA) and heparinase treated as described [19]. Primary MCT miRNA expression profiling was performed at the OSU Nucleic Acid Shared Resource using the TaqMan Array Human miRNA Panel (Human A Cards, v.2, Applied Biosystems, Foster City, CA, USA) as described previously [20]. This panel assays the expression of 377 human miRNAs, 151 of whose mature sequences are 100% conserved between human and dog (Sanger miRBase v.12). Raw data analysis, normalizer selection and statistical analysis were performed using the real-time PCR analysis software Statminer (Integromics, Madison, WI, USA). The snRNA U6 was confirmed to be stably expressed in our sample set and the mean used as the normalizer value. Relative gene expression was calculated using the comparative threshold cycle method [21]. Gene expression heat maps were generated using Treeview PC-based software [22].

### RNA isolation and quantitative real-time PCR

RNA was extracted from cell lines using TRIzol (Invitrogen) and real-time PCR was performed using the Applied Biosystems StepOne Plus Detection System. MiR-9 is highly conserved and shares 100% homology between dogs, humans, and mice. Mature miR-9 expression was performed using Taqman miRNA assays (Applied Biosystems). 50 ng total RNA was converted to first-strand cDNA with miRNA-specific primers, followed by real-time PCR with TaqMan probes. All samples were normalized to U6 snRNA.

Real-time PCR was performed to validate changes in mRNA expression for selected genes affected by miR-9 over expression. cDNA was made from 1 μg of total RNA using Superscript III (Invitrogen). CMA1, HSPE, IFITM3, MLANA, PERP, PPARG, PDZK1IP1, SERPINF1, SLPI, TLR7, CD200R1, CD200R4 and 18S transcripts were detected using Fast SYBR green PCR master mix (Applied Biosystems) according to the manufacturer’s protocol; primer sets are detailed in Table 1. Normalization was performed relative to 18S rRNA. All reactions were performed in triplicate and included no-template controls for each gene. Relative gene expression for all real-time PCR data was calculated using the comparative threshold cycle method [21]. Experiments were repeated 3 times using samples in triplicate.

**Table 1:** Primers for quantitative reverse transcriptase polymerase chain reaction

**Primers**	**Primer sequences**
Mouse Cma1 292F	5’-GAA GAC ACG TGG CAG AAG CTT GAG-3’
Mouse Cma1 521R	5’-GTG TCG GAG GCT GGC TCA TTC ACG-3’
Mouse Hspe F479	5’-GCT CAG TGG ACA TGC TCT ACA G-3’
Mouse Hspe R697	5’-GCA ACC CAT CGA TGA GAA TGT G-3’
Mouse Ifitm3 115F	5’-GCT TCT GTC AGA ACT ACT GTG-3’
Mouse Ifitm3 339R	5’-GAG GAC CAA GGT GCT GAT GTT CAG-3’
Mouse Mlana 125F	5’-GCT GCT GGT ACT GTA GAA GAC G-3’
Mouse Mlana 322R	5’-GTG AAG AGA GCT TCT CAT AGG CAG-3’
Mouse Pdzk1ip1 F520	5’-GTT CTG GCT GAT GAT CAC TTG ATT G-3’
Mouse Pdzk1ip1 R769	5’-GAT AGA AGC CAT AGC CAT TGC TG-3’
Mouse SerpinF1 712F	5’-GTG AGA GTC CCC ATG ATG TCA G-3’
Mouse SerpinF1 910R	5’-GTT CTC GGT CGA TGT CAT GAA TG-3’
Mouse Tlr7 F2284	5’-GTC ATT CAG AAG ACT AGC TTC CCA G-3’
Mouse Tlr7 R2441	5’-GTC ACA TCA GTG GCC AGG TAT G-3’
Mouse Cd200r1 659F	5’-GTA ACC AAT CTC TGT CCA TAG-3’
Mouse Cd200r1 902R	5’-GTC ACA GTA TCA TAG AGT GGA TTG-3’
Mouse Cd200r4 312F	5’-GCC TCC ACA CCT GAC CAC AG-3’
Mouse Cd200r4 532R	5’-GTC CAA GAG ATC TGT GCA GCA G-3’
Mouse Perp F108	5’-GCA GTC TAG CAA CCA CAT CCA G-3’
Mouse Perp R267	5’-GCA CAG GAT GAT AAA GCC ACA G-3’
Mouse Slpi F142	5’-GAG AAG CCA CAA TGC CGT ACT G-3’
Mouse Slpi R378	5’-GAC TTT CCC ACA TAT ACC CTC ACA G-3’
Mouse Pparg F682	5’-GAT ATC GAC CAG CTG AAC CCA G-3’
Mouse Pparg R983	5’-GCA TAC TCT GTG ATC TCT TGC ACG-3’
18S V2F	5’-AAA TCC TTT AAC GAG GAT CCA TT-3’
18S V2R	5’-AAT ATA CGC TAT TGG AGC TGG A-3’

### MiR-9 lentivirus infection

Lentiviral constructs were purchased from Systems Biosciences (Mountain View, CA, USA). Packaging of the lentiviral constructs was performed using the pPACKH1 Lentivector Packaging KIT (catalog no. LV500A-1) according to the manufacturer’s instructions. P815 and C57 mouse mastocytoma cells and mouse BMMCs (10^5^ cells) were transduced with empty lentivirus (catalog no. CD511B-1) or pre-miR-9-3 lentivirus (catalog no. PMIRH9-3PA-1). FACS-mediated cell sorting based on GFP expression was performed 72 hours post-transduction and miR-9 expression was evaluated by real-time PCR (Applied Biosystems).

### Transcriptional profiling of cells transduced with miR-9 lentivirus

RNA was extracted from mouse BMMCs and P815 cells transduced with empty lentivirus or pre-miR-9-3 lentivirus from three separate transduction experiments using TRIzol (Invitrogen). A secondary RNA cleanup step was performed using QIAGEN RNeasy Total RNA isolation kit (QIAGEN GmbH, Hilden, Germany) and RNA integrity was assessed using RNA 6000 Nano LabChip® Kits on the Agilent Bioanalyzer 2100 (Agilent Technologies, Palo Alto, CA, USA). RNA was labeled with Cy3 using RNA ligase and hybridized to GeneChip® Mouse Gene 2.0 ST Arrays (Affymetrix, Santa Clara, CA, USA). Ratios of signals were calculated and transcripts that were up-regulated or down-regulated by at least 2-fold were identified (p < 0.05). Data analysis, statistical analysis, and generation of gene expression heat maps were performed using Affymetrix® Transcriptome Analysis Console (TAC) Software. Prediction of miR-9 binding to the 3’-UTR of genes down-regulated by miR-9 was performed with computer-aided algorithms obtained from TargetScan (http://www.targetscan.org), PicTar (http://pictar.mdc-berlin.de), miRanda (http://www.microrna.org), and miRWalk (http://www.umm.uni-heidelberg.de/apps/zmf/mirwalk).

### Matrigel invasion assay

To assess the effect of miR-9 expression on invasion, cell culture inserts (8-μm pore size; Falcon) were coated with 100 μL of Matrigel (BD Bioscience, San Jose, CA, USA) to form a thin continuous layer and allowed to solidify at 37°C for 1 hour. P815 and C57 cell lines, and mouse BMMCs (5 × 10^5^/mL) transduced with control lentivirus or pre-miR-9-3 lentivirus were prepared in serum-free medium and seeded into each insert (upper chamber) and media containing 10% fetal bovine serum was placed in the lower chamber. The cells were incubated for 24 hours to permit invasion through the Matrigel layer. Cells remaining on the upper surface of the insert membrane were wiped away using a cotton swab, and cells that had migrated to the lower surface were stained with crystal violet and counted in ten independent 20× high powered fields for each sample. Experiments were repeated 3 times using samples in triplicate.

### Evaluation of proliferation and apoptosis

Changes in cell proliferation were assessed using the CyQUANT® Cell Proliferation Assay KIT (Molecular Probes, Eugene, OR, USA) as previously described [23]. P815 and C57 cells (15 × 10^4^) transduced with control lentivirus or pre-miR-9-3 lentivirus were seeded in 96-well plates for 24, 48, and 72 hours prior to analysis. Nontransduced P815 and C57 cells served as negative control wells. Fluorescence was measured using a SpectraMax microplate reader (Molecular Devices, Sunnyvale, CA, USA). Cell proliferation was calculated as a percentage of untransduced control cells.

Caspase-3/7 activity was determined using the SensoLyte® Homogeneous AMC Caspase- 3/7 Assay KIT (Anaspec Inc, San Jose, CA, USA) as previously described [24]. P815 and C57 cells (5.0 × 10^4^) transduced with either empty lentivirus or pre-miR-9-3 lentivirus were plated for 24 and 48 hours in 96-well plates prior to analysis. Fluorescence was measured on a SpectraMax microplate reader (Molecular Devices). Levels of caspase 3/7 activity were reported after subtraction of fluorescence levels of wells with medium only.

### Statistical analysis

Statistical analysis relative to miRNA expression data was performed with Statminer software (Integromics) and p-values of <0.05 were considered statistically significant. Statistical analysis relative to mRNA expression data was performed using Affymetrix® Transcriptome Analysis Console (TAC) Software. Differential gene expression was determined by one-way ANOVA comparison test and p-values of <0.05 were considered statistically significant. All experiments with the exception of those involving canine BMMCs were performed in triplicate and repeated 3 times. Experiments using canine BMMCs were performed in triplicate, but repeated only twice because of limited cell numbers. Data were presented as mean plus or minus standard deviation. The difference between two group means was analyzed using the Students *t*-test and a one-way analysis of variance (ANOVA) was performed for multiple variable comparisons. P-values of <0.05 were considered significant.

## Results

### MiRNA expression in primary canine MCTs is associated with biological behavior

**Figure 1:**
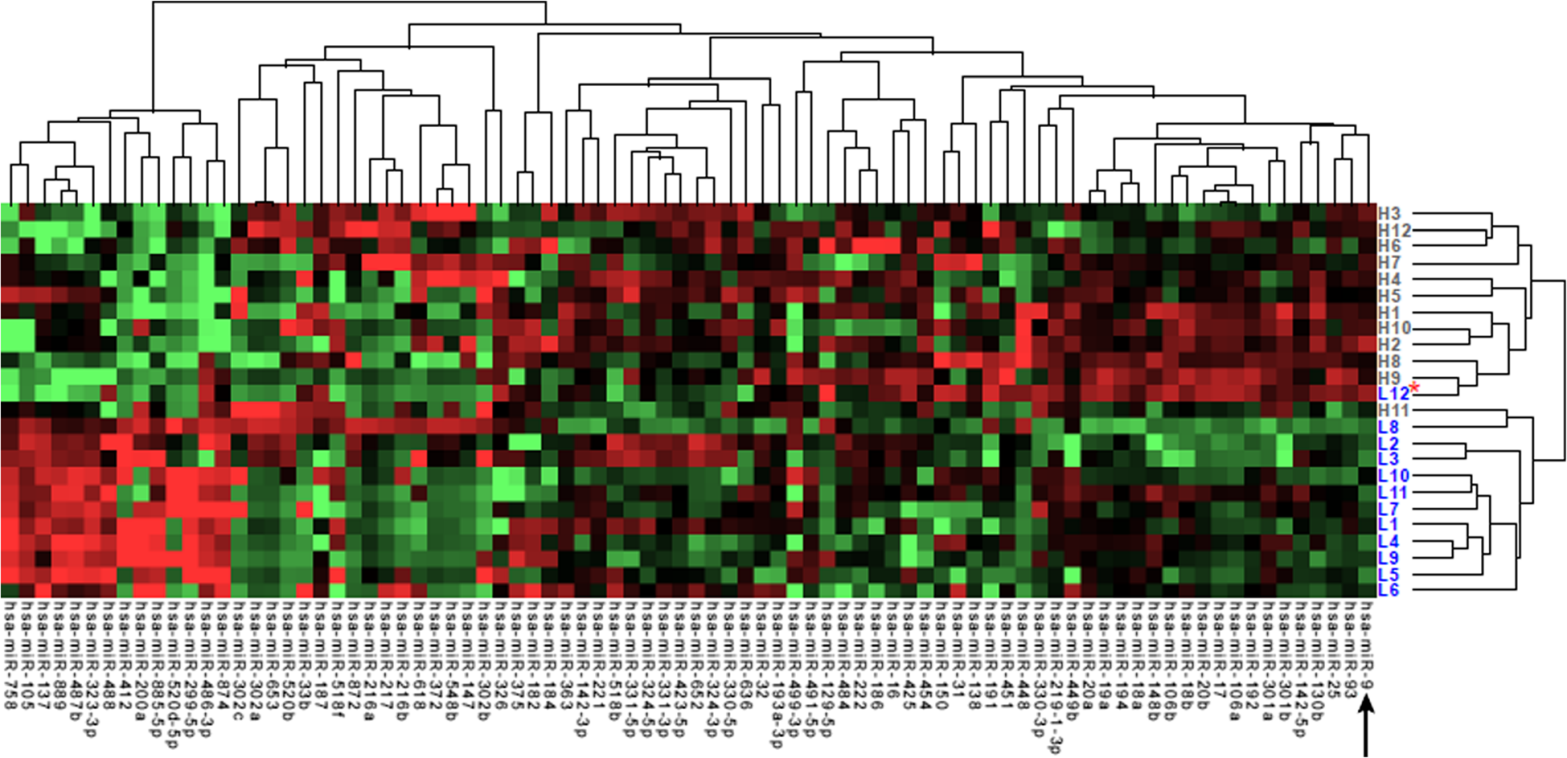
**MiRNA expression in primary canine MCTs is associated with biological behavior.** Primary canine MCTs were obtained from dogs diagnosed with benign tumors (n = 12) or biologically high grade metastatic tumors (n = 12). Real-time PCR profiling was performed using Applied Biosystems Human TaqMan Low Density miRNA Arrays to assess mature miRNA expression in primary tumors. Unsupervised hierarchical cluster analysis separated samples into two groups based on biological behavior and demonstrate unique miRNA expression profiles associated with biologically low-grade (L) tumors or high-grade (H) tumors (P < 0.05). (*) indicates primary tumor sample from a dog with a benign mast cell tumor that clustered with the biologically high grade MCT group.

To investigate the role of miRNA dysregulation in the biologic behavior of mast cell disease, global miRNA expression in primary canine MCTs obtained from 24 dogs diagnosed with benign tumors (n = 12) or with biologically high-grade tumors (n = 12) was evaluated using real-time PCR-based TaqMan Low Density miRNA Arrays (Applied Biosystems). An unsupervised hierarchial cluster analysis of all primary MCTs readily separated tumors into groups based on biological behavior with aggressive, highly metastatic MCTs clustering together and clinically benign MCTs clustering together separately (Figure 1). We identified 45 miRNAs that had significantly higher expression in biologically high-grade MCTs compared to biologically low-grade MCTs, while 7 miRNAs had lower expression (Table 2). These data demonstrate that biologically high-grade and low-grade canine MCTs possess distinct miRNA expression signatures.

**Table 2:** MiRNA signature associated with biologically high-grade MCTs

**miRNA**	**Fold-change**	**p-value**	**miRNA**	**Fold-change**	**p-value**
	**Gene expression**			**Gene expression**	
	**High vs low grade MCT**			**High vs low grade MCT**	
Upregulated miRNAs					
hsa-miR-301b	4.2	0.00022	hsa-miR-520b	1.8	1.8
hsa-miR-454	2.4	0.00032	hsa-miR-216b	4.6	0.023
hsa-miR-9	3.2	0.0010	hsa-miR-302b	3.2	0.024
hsa-miR-147	3.9	0.0017	hsa-miR-106b	1.6	0.026
hsa-miR-138	2.5	0.0022	hsa-miR-618	3.0	0.027
hsa-miR-330-5p	3.1	0.0027	hsa-miR-518f	3.2	0.029
hsa-miR-187	5.1	0.0029	hsa-miR-182	2.8	0.030
hsa-miR-106a	2.1	0.0044	hsa-miR-142-5p	1.7	0.031
hsa-miR-636	2.7	0.0052	hsa-miR-301a	2.8	0.032
hsa-miR-17	2.0	0.0057	hsa-miR-217	3.9	0.033
hsa-miR-449b	3.2	0.0069	hsa-miR-652	2.0	0.039
hsa-miR-130b	2.2	0.0082	hsa-miR-186	1.5	0.039
hsa-miR-192	2.5	0.0095	hsa-miR-19a	1.8	0.040
hsa-miR-448	3.1	0.010	hsa-miR-872	1.5	0.041
hsa-miR-425	3.0	0.011	hsa-miR-148b	1.8	0.043
hsa-miR-193a-3p	2.6	0.011	hsa-miR-451	2.4	0.044
hsa-miR-18b	2.2	0.014	hsa-miR-423-5p	1.7	0.048
hsa-miR-93	2.1	0.014	hsa-miR-191	1.5	0.049
hsa-miR-548b-5p	2.3	0.015	Downregulated miRNAs		
hsa-miR-25	2.1	0.015	hsa-miR-885-5p	-4.2	0.00011
hsa-miR-324-3p	2.3	0.017	hsa-miR-874	-5.8	0.00018
hsa-miR-326	2.6	0.017	hsa-miR-486-3p	-4.6	0.00040
hsa-miR-18a	3.1	0.017	hsa-miR-299-5p	-4.2	0.0020
hsa-miR-20b	2.0	0.017	hsa-miR-488	-3.9	0.0063
hsa-miR-194	2.8	0.019	hsa-miR-200a	-5.5	0.034
hsa-miR-372	2.4	0.019	hsa-miR-412	-2.8	0.035

### miR-9 is overexpressed in biologically high-grade canine MCTs

**Figure 2:**
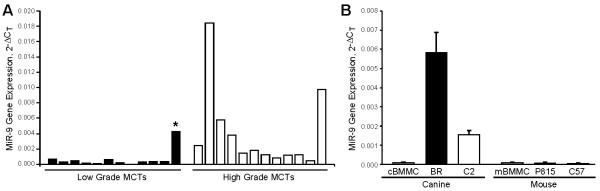
**MiR-9 is highly expressed in biologically high grade canine MCTs and malignant mast cell lines. (A)** Real-time PCR evaluating mature miR-9 expression in primary canine MCTs demonstrated that the mean expression of miR-9 was 3.2-fold higher in aggressive, high grade MCTs compared to benign MCTs (*p* = 0.001). (*) indicates primary tumor sample from a dog with a low-grade mast cell tumor that expressed high levels of miR-9 but had lymph node metastasis at the time of surgery. **(B)** Malignant canine BR and C2 mast cells, normal canine and mouse BMMCs, and malignant mouse C57 and P815 cells were cultured and real-time PCR was performed to assess miR-9 expression levels. Three independent experiments were performed and all reactions were performed in triplicate. The experiments were repeated 3 times in the cell lines and twice for normal cBMMCs.

The miRNA array performed above identified miR-9 as overexpressed in MCTs that metastasized and resulted in death of affected dogs. This finding was confirmed by real-time PCR in which a 3.2-fold increase in miR-9 expression was identified in biologically aggressive MCTs as compared to benign MCTs (Figure 2A). Furthermore, miR-9 expression correlates with tumor grade and metastatic status in human breast cancer, providing further support for the idea that altered miR-9 expression may be an important regulator of aggressive biological behavior in MCTs (33). Interestingly, one of the primary tumor samples collected from a dog with a biologically low-grade MCT expressed high levels of miR-9 and the unsupervised hierarchial clustering of all 24 MCTs demonstrated that this dog’s tumor clustered with the biologically high-grade tumors (Figure 1). Clinical data was subsequently reviewed for all dogs and it was determined that this dog had histopathologically confirmed evidence of metastatic mast cells present in a regional lymph node surgically excised at the time of primary tumor removal. Additionally, one high-grade MCT clustered with the low-grade tumors, however, this may have been due, in part, to variations in stroma/inflammatory cells within the primary tumor specimen or baseline necrosis within the tumor that influenced the proportion of tumor cells. Taken together, these findings suggest a correlation between miR-9 expression levels in primary canine MCTs and metastatic behavior.

### miR-9 expression is up-regulated in canine malignant mast cell lines

Given the potential link between miR-9 expression and biological behavior of MCTs, we next evaluated miR-9 expression in canine (BR and C2) and murine (C57 and P815) mast cell lines and normal canine and murine BMMCs by real-time PCR. As shown in Figure 2B, canine mastocytoma cells exhibited higher levels of miR-9 expression when compared with normal canine BMMCs. In contrast, both mouse C57 and P815 cells and mouse BMMCs demonstrated low basal levels of miR-9. The mouse P815 mastocytoma cell line is a leukemia of mast cell origin, whereas the canine BR and C2 mastocytoma cells are derived from cutaneous tumors. The differences in the biology of these diseases may account for the observed differences in miR-9 expression in canine and murine cell lines. Low miR-9 expression in P815 cells may reflect the fact that these cells represent a true leukemia, in contrast to the BR and C2 cell lines which are derived from cutaneous tumors that would metastasize via the lymphatic system. Given prior work from our laboratory showing that the C2 line exhibits invasive behavior *in vitro* while the P815 line does not [24], it was possible that miR-9 expression was associated with the invasive behavior of mast cells.

### Overexpression of pre-miR-9 enhances invasion of malignant mast cell lines

**Figure 3:**
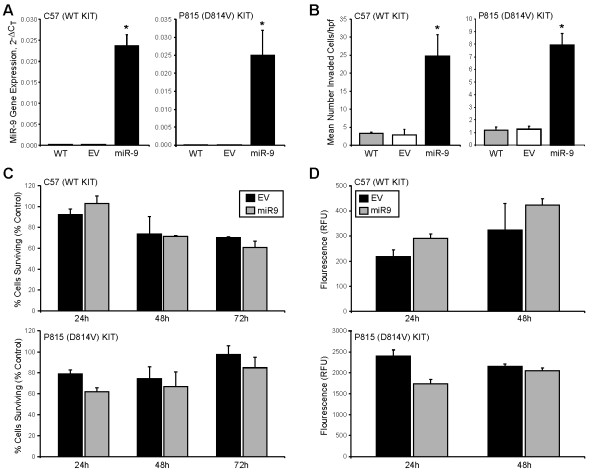
**Overexpression of miR-9 enhances invasion of malignant mast cells and has no effect on cell proliferation or apoptosis. (A)** Mouse P815 and C57 mast cells transduced with pre-miR-9-3 lentivirus or empty vector control were sorted to greater than 95% purity based on GFP expression. MiR-9 levels were assessed by real-time PCR in wild-type, empty vector, and miR-9 expressing cells (*p < 0.05). Three independent experiments were performed and all reactions were performed in triplicate. **(B)** Mouse P185 and C57 mast cells transduced with either empty vector or pre-miR-9-3 lentivirus were transferred onto cell culture inserts coated with Matrigel® for 24 hrs. After incubation, membranes were stained and cells that had invaded the membrane were counted in ten independent 20x hpf for each sample. Three independent experiments were performed and all assays were performed in triplicate wells (*p < 0.05). **(C)** Mouse P185 and C57 mast cells were transduced with either empty vector or pre-miR-9-3 lentivirus vector and cell proliferation was analyzed at 24, 48, and 72 hours using the CyQUANT method. Nontransduced P815 and C57 cells served as non-treated controls. Three independent experiments were performed and all samples were seeded in triplicate wells. Values are reported as percentage of untransduced control cells. **(D)** Mouse P185 and C57 mast cells transduced with either empty vector or pre-miR-9-3 lentivirus were assessed for apoptosis at 24 and 48 hours by measuring active caspase-3/7 using the SensoLyte® Homogeneous AMC Caspase-3/7 Assay kit. Relative fluorescence units are reported after subtraction of fluorescence levels of wells with medium only.

To investigate the functional consequences of miR-9 overexpression in malignant mast cell lines, we stably expressed miR-9 in the mouse P815 and C57 cell lines that exhibit low basal levels of this miRNA using an empty or pre-miR-9-3 expressing lentivirus vector. Following transduction, GFP + cells were sorted and miR-9 expression was confirmed by real-time PCR (Figure 3A). The invasive capacity of cells was then evaluated using a standard Matrigel invasion assay after 24 hours of culture. As shown in Figure 3B, enforced expression of miR-9 in C57 and P815 mast cell lines significantly enhanced their invasion compared to cells expressing empty vector.

### miR-9 has no effect on cell proliferation or caspase-3,7 dependent apoptosis in malignant mast cells

To investigate whether overexpression of miR-9 in malignant mast cells affected their capacity to proliferate or survive, mouse C57 and P815 cell lines expressing pre-miR-9-3 lentivirus or empty vector control were cultured for 24, 48, and 72 hrs and the impact on cell proliferation and apoptosis was assessed. No effects of miR-9 on proliferation or apoptosis were observed in either cell line when compared to cells expressing empty vector (Figure 3C and D).

### miR-9 expression enhances invasion in normal mouse BMMCs

**Figure 4:**
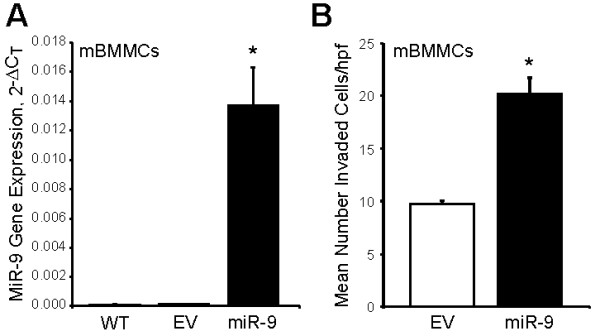
**Overexpression of miR-9 enhances invasion in normal mouse bone marrow-derived mast cells. (A)** Normal mBMMCs transduced with pre-miR-9-3 lentivirus or empty vector control were sorted to greater than 95% purity based on GFP expression. MiR-9 levels were assessed by real-time PCR (*p < 0.05). Three independent experiments were performed and all reactions were performed in triplicate. **(B)** mBMMCs transduced with either empty vector or pre-miR-9-3 lentivirus were transferred onto cell culture inserts coated with Matrigel® for 24 hrs. After incubation, cells remaining on the upper surface of the insert membrane were wiped away using a cotton swab, and cells that had migrated to the lower surface were stained with crystal violet and counted in ten independent 20x hpf for each sample. Three independent experiments were performed and all samples were performed in triplicate wells (*p < 0.05).

To characterize the biological consequences of miR-9 overexpression in normal mast cells, we transduced murine BMMCs with pre-miR-9-3 lentivirus or empty control vector. MiR-9 overexpression in transformed BMMCs was confirmed by quantitative real-time PCR (Figure 4A). To assess the effect of ectopic miR-9 expression on the invasive capacity the BMMCs, a Matrigel invasion assay was again performed. Consistent with findings in the P815 and C57 cell lines, enforced expression of miR-9 in mouse BMMCs significantly enhanced their invasive capacity compared to cells expressing empty vector (Figure 4B). Together, these data suggest that miR-9 promotes an invasive phenotype in mast cells.

### Microarray analysis identified genes affected by miR-9

**Figure 5:**
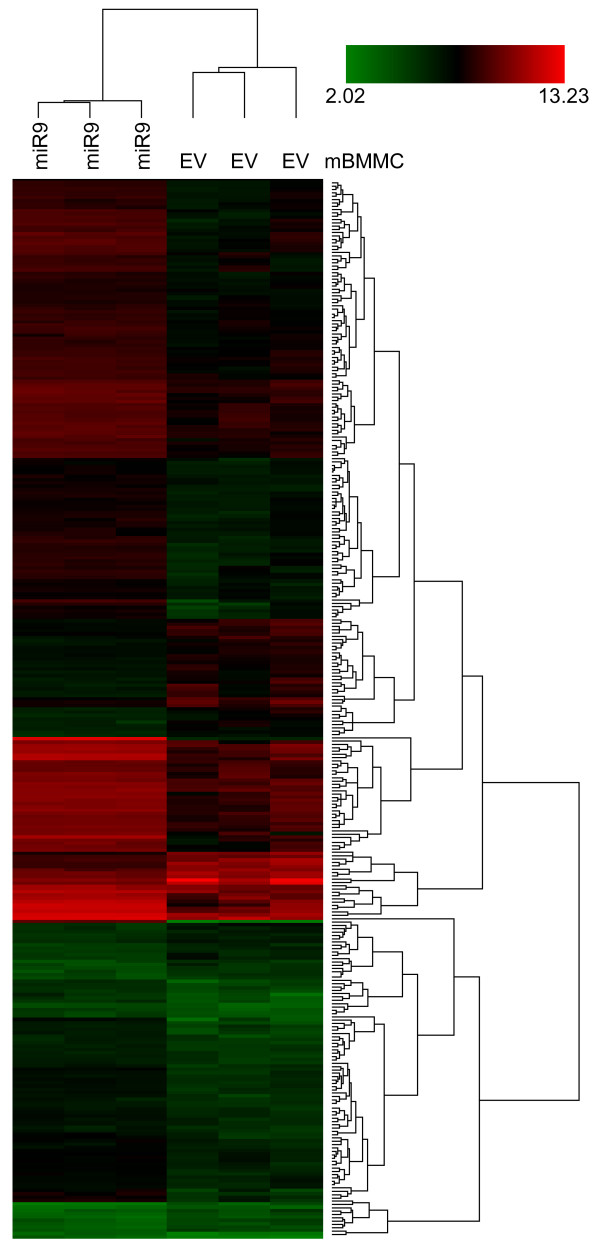
**Overexpression of miR-9 in normal mouse bone marrow-derived mast cells significantly alters gene expression.** Normal mBMMCs transduced with pre-miR-9-3 lentivirus or empty vector control were sorted based on GFP expression. RNA was harvested from mouse BMMCs transduced with empty vector or pre-miR-9-3 lentivirus from three separate transduction experiments. Transcriptional profiling was performed using Affymetrix GeneChip® Mouse Gene 2.0 ST Arrays. Hierarchical clustering was performed for 450 genes differentially expressed (p < 0.05) in mBMMCs expressing either empty vector (EV) or miR-9 (miR9) as determined by one-way ANOVA comparison test (p < 0.05). Mean centered signal intensities of gene-expression are depicted by the log2 of the ratio of the signals against the average signal for each comparison. Color areas indicate relative expression of each gene after log2 transformation with respect to the gene median expression (red above, green below, and black equal to the mean).

**Figure 6:**
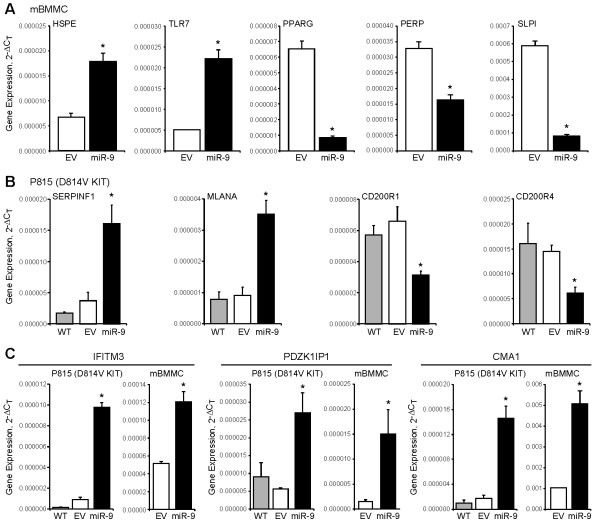
**Identification of transcripts dysregulated by miR-9 overexpression in normal murine BMMCs and P815 malignant mast cells. (A)** Transcriptional profiling of mBMMCs expressing pre-miR-9-3 lentivirus or empty vector control was performed using Affymetrix GeneChip® Mouse Gene 2.0 ST Arrays to identify genes showing differential expression (>2-fold) with miR-9 overexpression. Real-time PCR was performed to validate changes in gene expression for transcripts (HSPE, TLR7, PERP, PPARG, SLPI) altered by miR-9 overexpression in mBMMCs (*p < 0.05). **(B)** Transcriptional profiling of P815 mast cells expressing pre-miR-9-3 lentivirus or empty vector control was performed as described above. Real-time PCR was performed to independently validate expression levels of genes (SERPINF1, MLANA, CD200R1, CD200R4) altered by enforced miR-9 expression in P815 cells (*p < 0.05). **(C)** Mouse BMMCs and P815 cells expressing pre-miR-9-3 lentivirus or empty vector control were collected and real-time PCR for IFITM3, PDZK1IP1, and CMA1 was performed (*p < 0.05). Three independent experiments were performed using cells from 3 separate transduction experiments and all reactions were performed in triplicate.

To gain insight into possible mechanisms underlying the observed miR-9-dependent invasive behavior of mast cells, we compared the transcriptional profiles of murine BMMCs overexpressing miR-9 to those expressing empty vector and found marked changes in gene expression (Figure 5). In BMMCs overexpressing miR-9, 321 transcripts were significantly up-regulated (>2-fold) and 129 transcripts were significantly down-regulated (Table 3, Table 4). Bioinformatic analysis identified putative miR-9 target sites within the 3’-UTR of 40 gene transcripts that were significantly down-regulated with miR-9 overexpression, suggesting that miR-9 may directly target and regulate expression of these candidate genes (Table 3, bolded). Real time PCR confirmed that one of these genes, peroxisome proliferator-activated receptor δ (PPARG) was down-regulated, a finding consistent with recent studies demonstrating regulation of PPARG by miR-9 through direct targeting of its 3’-UTR [25]. We performed real-time PCR to validate changes in gene expression for several transcripts altered by miR-9 overexpression in BMMCs. Consistent with our microarray results, we found that transcripts for HSPE and TLR7 were significantly up-regulated in BMMCs expressing miR-9, whereas transcripts for PPARG, PERP, and SLPI were significantly down-regulated compared to empty vector controls (Figure 6A).

**Table 3:** Gene transcripts altered by miR-9 overexpression in BMMCs

**Downregulated with miR-9 expression (BMMCs)**		
1-Sep	Ell2	Phgdh
**1300014I06Rik**	**Emp1**	**Pi16**
**1600029D21Rik**	Eya2	Plk2
2810025M15Rik	Fn1	**Plod2**
5830428M24Rik	**Fzd4**	Ppap2b
A2ld1	Gatm	**Pparg**
Akr1c18	**Glrp1**	Ppic
Alox15	Gm10021	Prg2
**Amigo2**	Gm19524	Prss34
**Ankrd22**	Gm2663	Psat1, LOC100047252
Ankrd55	Gm6445	**Rbp4**
Arfip1	**Gnpnat1**	Reep6
Arg2	Gpc4	Retnla
**Asb2**	**Gpt2**	**Rhoj**
Asns	Grb10	**Scd1**
**Atp1b1**	**H2-M2**	Scn7a
Atp8b4	Hal	**Serpinb9b**
Awat1	Hdc	Sgce
BC100530	**Hgf**	**Slamf1**
Bex1	Il18rap	Slc16a1
Bri3bp	Il1f9	**Slc22a3**
C87414	Il6st	Slc36a4
Ccdc88c	Itk	**Slc43a3**
Ccl17	Klf5	**Slc7a1**
Ccl24	Klrb1f	Slc7a5
**Ccl8**	Lama5	Slpi
Cd209d	Lcn2	Snord70
Cd24a	LOC100861767	Speer4e, Gm17019
Cd36	LOC100862026	Stfa2
**Cdh17**	Lrrk2	Stfa2l1
Cdkn2b	**Mbnl3**	**Sulf2**
**Celsr1**	Mcpt8	**Syne1**
Chi3l4	Mgam	Taf1d
Clec4e	**Mmp13**	**Tfrc**
**Colec12**	Mrgpra6	**Thbs1**
Csf3r	Niacr1	Tm4sf19
Ctsg	Nrg1	**Tmem26**
Ctsk	O3far1	Tnfrsf10b
**Ctsl**	**Olr1**	**Tspan7**
Dennd2d, 2010016I18Rik	Pdlim1	Ube2e2
Dnajc6	Perp	Vmn1r129
**Ear2, Ear12, Ear3**	Pga5	Zbtb10
**Egln3**	Phf10	Zfp608

Bold indicates predicted miR-9 targets.

**Table 4:** Gene transcripts altered by miR-9 overexpression in BMMCs

**Upregulated with miR-9 expression (BMMCs)**			
1810011H11Rik	Ddx60	Irg1	Plxna1
2310028H24Rik	Dnaja4	Itgb5	Plxnb3
3110043O21Rik	Dpep2	Kcnab3	Plxnc1
4930420K17Rik	Dusp22	Kcne3	Ppargc1a
5033411D12Rik	E130215H24Rik	Kctd12	Ppfibp2
5430435G22Rik	E330020D12Rik	Kctd6	Ppp1r14c
6330415B21Rik	Ednra	Khdc1a	Prdx1, LOC100862012
9030625A04Rik	Egr1	Kit	Prickle1
9430070O13Rik	Emx2	Klf2	Psd3
9930111J21Rik2	Epsti1	Klk1b1	Psg23
A130040M12Rik	Esco2	Klk1b11	Ptafr
A230098N10Rik	Esr1	Klk1b27	Ptger2
A430084P05Rik	Evl	Klk1b5	Ptplad2
A4galt	F13a1	Kmo	Ptpn13
Abi3	Fabp5	Lce6a	Qpct
Adamtsl3	Fabp5, Gm3601	LOC100038947	Rasgrp3
Adrb2	Fam125b	LOC100861753	Rassf4
AI593442	Fam55d	LOC100861977	Rbm47
AI607873	Fam69a	LOC100862646	Rin2
Alcam	Fcgr4	Lphn1	Rnase4, Ang
Alpk2	Fkbp1b	Lrp1	Rnase6
Ank	Fos	Lrrc16a	Rnf180
Ano3	Fpr2	Lrrc25	Rny1
Aoah	Galnt10	Lrrtm1	Rps6ka2
Apobec1	Galntl4	Ltf	Rsph9
Ar	Gas6	Ly6i	Rtp4
Arhgap20	Gbp3	Lyz1	Ryr3
Arhgap24	Gbp4	Maf	Scn1b
Arhgap31	Gbp5	Mast4	Scpep1
Arl5b	Gbp8	Mc1r	Serpinb8
Asphd2	Gbp9	Mecom	Siglec1
Bank1	Gcet2	Mgl2	Sirpb1a
BC013712	Gdf15	Mgll	Sirpb1b
Bcl2a1b, Bcl2a1a	Gdpd1	Mir15b	Slc30a2
Bcl2a1d, Bcl2a1a, Bcl2a1b	Ggh	Mir181a-1	Slc37a2
Bhlhe41	Glul	Mir3095	Slc39a4
Bmpr2, Gm20272	Gm11711, Cd300lh	Mir3108	Slc40a1
Bst1	Gm12250	Mir511	Slc4a11
Bst2	Gm14446	Mir701	Slc6a12
C1qb	Gm15915	Mlph	Slc9a9
C1qc	Gm1673	Mmp2	Slfn5
C330018A13Rik	Gm1966	Mnda, Ifi204	Smpdl3b
C5ar1	Gm20099	Mpeg1	Smpx
Cacnb4	Gm4759	Mrgpra9	Snord14e, Hspa8
Cadm3	Gm4951	Mrgprb2	St3gal5
Car8	Gm5431	Ms4a4a	St6galnac3
Ccl2	Gm7977	Ms4a6b	Stab1
Ccl4	Gmpr	Ms4a6c	Stfa3
Ccl7	Gna14	Ms4a6d	Sult1a1
Ccnd1	Gp1ba	Ms4a7	Syn2
Ccr1l1	Gp5	Msr1	Syngr1
Ccr3	Gpm6a	Mtss1	Tdrd5
Ccr5	Gpr55	Nav1	Tek
Ccrl2	Grap2	Neb	Tgfbr2
Cd14	H2-DMa	Nlrp1b	Tlr1
Cd180	H2-DMb2	Nlrp1c	Tlr13
Cd200r2	H2-Q6,H2-Q8,LOC68395	Npy1r	Tlr7
Cd28	Hey2	Nrn1	Tlr9
Cd300a	Hist1h1d	Oas2	Tmem106a
Cd300lb	Hist1h1e	Oasl2	Tmem233
Cd300ld	Hist1h2bg	Olfr1033	Tmem86a
Cd86	Hist2h3b	Olfr110	Tnfrsf1b
Cdh2	Hist2h4	Olfr111	Tns1
Chst15, Gm10584	Hist3h2a	Olfr1392	Trem1
Cited4	Hist4h4	Olfr1393	Trim30c
Clec4a1	Hivep2	Olfr915	Trim30d
Clec4d	Hpse	Olfr916	Trim58
Clec4n	Hsd3b6	Olfr917	Trpc6
Cma1	Ier2	Olfr918	Tsc22d3
Cma2	Ifi204	Orm3	Tspan13
Cmklr1	Ifi27l2a, Ifi27l2b	P2rx7	Tspan8
Creb5	Ifitm3	P2ry6	Tubb2b
Csf1r	Ifitm6	Pcdhga10	Txk
Ctnna2	Ighm	Pcdhgb6	Ugt1a10
Ctsh	Igk-V28	Pdzk1ip1	Unc93b1
Cx3cr1	Il18	Pgap1	Zbp1
Cybb	Il2ra	Pid1	Zbtb8a
Cyp4a12a	Il6ra	Pion	Zfhx3
Dab2	Iqsec3	Pld2	
Darc	Irf5, Tnpo3	Pld4	
Dbc1	Irf8	Plekhm3	

Similar transcriptional profile analysis was performed using malignant mouse P815 cells and we identified 46 transcripts significantly up-regulated (>2-fold) and 48 transcripts significantly down-regulated in the miR-9 expressing P815 cells (Table 5). Bioinformatic analysis identified putative miR-9 target sites within the 3’-UTR of 15 gene transcripts that were significantly down-regulated following miR-9 overexpression, suggesting that miR-9 may directly regulate these genes (Table 5, bolded). Real-time PCR demonstrated that expression of SERPINF1 and MLANA transcript was up-regulated in P815 cells overexpressing miR-9, whereas CD200R1 and CD200R4 was down-regulated compared to empty vector controls (Figure 6B).

**Table 5:** Gene transcripts altered by miR-9 overexpression in P815 mast cells

**Upregulated with miR-9 expression (P815)**	**Downregulated with miR-9 expression (P815)**
Ifitm3	Ligp1
Pdzk1ip1	Ppm1j
Cma1	Gbp2
Pfkp	Hist2h3c1
Serpinf1	Ly6a
Trim63	**Cd200r1**
As3mt	Gzmb
Speg	**Gbp6**
Mlana	Afp
Mgl1	**Ifit1**
Tmem223	Parp14
Fjx1	Ctla2a
Vamp5	Igtp
Cthrc1	**Slamf1**
Ptgis	Tnfrsf9
Ass1	Cpa3
Ahi1	Ctla2b
Akap13	**Tgtp//Tgtp2**
Prf1	**Rabgap1l**
Ston2	Clec4e
Hcfc1	Parp9
Trak1	**Plekha1**
Ankrd6	Il1rl1
Atn1///Rnu7	Sdf2l1
Fam122b	Gvin1
Mll1	**Il2ra**
Zbtb12	**Fcgr1**
Ahnak	Gfi1
Sec14l1	**Thoc1**
Mknk2	Hist1h2ad
Apobec2	Tmed7
Tspan32	**Ugt1a1**
Hnrnpl	Taf7l
Serbp1	Slc13a2
Msi2	Cd200r4
Myl9	Vegfc
Runx2	Oasl2
Gstm1	**Socs3**
Epb4.1l4b	677168///Isg15
LOC100041694	**Ctso**
2310051F07Rik	Adam8
Arx///LOC100044440	Samd9l
Mest	1810014B01Rik
Mpp4	LOC641050
Rp131	Lrrc28
Sphk1	**Hist2h2be**
	Ebi3
	**Igf1**

Bold indicates predicted miR-9 targets.

A comparison of the transcriptional profiles both from normal BMMCs and malignant P815 cells overexpressing miR-9 found that most gene transcripts altered by miR-9 were specific to normal or malignant mast cells. We identified 7 gene transcripts (IFITM3, PDZK1IP1, CMA1, MGL1, TMEM223, SLAMF1, CLEC4E) that showed similar changes in expression following miR-9 overexpression in both BMMCs and P815 cells. We performed real-time PCR to validate changes in gene expression for several transcripts altered by miR-9 overexpression, including mast cell chymase (CMA1), interferon-induced transmembrane protein 3 (IFITM3), and PDZK1 interacting protein 1 (PDZK1IP1). Consistent with our microarray results, real-time PCR confirmed that enforced miR-9 expression significantly upregulated CMA1, IFITM3, and PDZK1IP1 transcripts in mouse BMMCs and P815 cells (Figure 6C). These findings provide further support for the notion that miR-9 induces alterations in gene expression that may contribute to the development of an invasive phenotype.

## Discussion

MiRNAs regulate various biological functions in normal cells such as growth and differentiation, and they are increasingly recognized as playing critical roles in cancer development and progression. Dysregulation of miRNA expression resulting from amplification or loss of miRNAs in tumors compared to their normal tissue counterparts suggests that miRNAs can function as either oncogenes or tumor suppressor genes [13]. Studies evaluating miRNA expression in spontaneously occurring tumors in dogs demonstrate that similar to human cancers, alteration of miRNAs likely contributes to tumorigenesis and that high-throughput methodologies used for the study of miRNAs in human tissues can also be applied to dogs [26-32].

Cutaneous MCTs are the most common skin tumor in dogs; however, little is known regarding mechanisms underlying malignant transformation of these cells. The biological behavior of canine MCTs ranges from relatively benign disease cured with surgical removal to aggressive, highly metastatic tumors ultimately resulting in the death of affected dogs. While the presence of activating *KIT* mutations helps to explain the behavior of some canine MCTs, little is known regarding the potential role of miRNAs in both normal and malignant mast cells. The purpose of this study was to begin to investigate the potential role of miRNA dysregulation in canine MCTs that exhibit aggressive biologic behavior.

MiRNA expression profiling of primary canine MCTs identified unique miRNA signatures associated with aggressive MCTs as compared to benign MCTs. The unsupervised hierarchical clustering of primary cutaneous MCTs based on their miRNA expression profiles recapitulated the grouping of the tumors based on their biological behavior, supporting the notion that miRNA dysregulation is associated with the biologic behavior of canine MCTs. Furthermore, we found that miR-9 expression was significantly upregulated in aggressive MCTs compared to benign MCTs. Interestingly, miR-9 was identified as a pro-metastatic miRNA in human breast cancer cell lines through its ability to enhance cell motility and invasiveness *in vitro* and metastasis formation *in vivo*[33]. More recently, miR-9 expression was found to be significantly increased in paired primary tumors and distant metastatic sites, suggesting direct involvement of miR-9 in the metastatic process [34,35]. In concordance with the potential role of miR-9 in malignant mast cell behavior, the BR and C2 canine malignant cell lines expressed high levels of miR-9 compared to normal canine BMMCs. Taken together, these data support the notion that dysregulation of miR-9 may contribute to the aggressive biologic behavior of some canine MCTs.

While activating *KIT* mutations clearly contribute to the malignant behavior of mast cells, additional cooperating or initiating genetic defects may be required for the malignant transformation and promotion of the metastatic phenotype [3]. Our data demonstrate that overexpression of miR-9 in the C57 and P815 mouse malignant mast cell lines and normal mouse BMMCs significantly enhanced the invasive behavior of mast cells and indicate that miR-9 induces a pattern of gene dysfunction associated with an invasive phenotype regardless of *KIT* mutation status.

While some studies have shown that miR-9 promotes metastasis formation [33,36-39] other contrasting studies suggest that increased expression of miR-9 suppresses metastasis formation [40,41] and that miR-9 inhibits tumor growth [42]. The opposing roles of miR-9 in various tissues may be explained by the expression of different mRNA targets in distinct cellular and developmental contexts. Indeed, miRNA effects do appear to be cell type/tissue specific and contextual in nature. Previous studies have demonstrated that miR-9 is overexpressed in CDX2-negative primary gastric cancers and miR-9 knockdown inhibits proliferation of human gastric cancer cell lines [43]. In contrast, miR-9 is downregulated in human ovarian tumor cells and overexpression of miR-9 suppresses their proliferation, in part by downregulating NFκB1 [40,42]. Moreover, miRNA dysregulation may affect only certain aspects of cell behavior. In our studies, miR-9 expression in mast cell lines did not provide a survival advantage or prevent apoptosis, but it did alter the invasive phenotype, supporting the contextual nature of miR-9 induced effects.

To gain insight into possible mechanisms underlying the observed miR-9-dependent invasive behavior of mast cells, we evaluated the effects of miR-9 expression on the transcriptional profiles of BMMCs and P815 cells. MiR-9 modulated the expression of a large number of gene transcripts, including down-regulation of several putative miR-9 targets identified by computational prediction programs. Furthermore, down-regulation of peroxisome proliferator-activated receptor δ (PPARG) was observed in BMMCs following enforced miR-9 expression, a finding consistent with recent studies demonstrating that regulation of PPARG expression is mediated by miR-9 through direct targeting of its 3’-UTR [25]. To draw firm conclusions regarding direct regulation of target gene expression by miR-9, a functional approach for each gene would be required to validate whether these genes are true miR-9 targets, which although relevant, was outside the scope of this study.

Overexpression of miR-9 significantly altered gene expression in both BMMCs and P815 cells, however, most gene transcripts affected by miR-9 expression differed between normal and malignant mast cells. These observed differences likely reflect variations in the impact of miR-9 that are dependent on cellular context. In our study, we identified gene transcripts that showed similar changes in expression following miR-9 overexpression in both normal and malignant mast cells and validated several genes demonstrating significant changes in expression (interferon-induced transmembrane protein protein 3, IFITM3; PDZK1 interacting protein 1, PDZK1IP1) or implicated in promoting the metastatic phenotype (mast cell chymase, CMA1). IFITM3 belongs to a family of interferon-induced transmembrane proteins that contribute to diverse biological processes, such as antiviral immunity, germ cell homing and maturation, and bone mineralization. The function of these proteins in mast cells is currently unclear [44]. PDZK1IP1 is a small, non-gycosylated membrane-associated protein that localizes to the plasma membrane and Golgi apparatus. While the function of PDZK1IP1 has not been evaluated in mast cells, overexpression of PDZK1IP1 has been documented in human ovarian, breast, and prostate carcinomas and this strongly correlates with tumor progression [45,46]. Furthermore, overexpression of PDKZK1IP1 in melanoma cell lines enhances cell proliferation, decreases apoptosis, increases cell migration and is, in part, mediated by an increase in reactive oxygen species (ROS) production [47].

Chymases are serine proteases possessing chymotrypsin-like activity expressed exclusively by mast cells that promote matrix destruction, tissue remodeling and modulation of immune responses by hydrolyzing chemokines and cytokines [48]. Given the role of chymase in the activation of matrix metalloproteases and extracellular matrix degradation, our findings suggest that miR-9 enhances invasion, in part, through increased expression chymase. Indeed, miR-9 overexpression in normal mast cells resulted in increased expression of CMA1 with a concomitant decrease in the expression of secretory leukocyte peptidase inhibitor (SLPI), a direct inhibitor of chymase [49]. These findings are consistent with the notion that that miR-9 promotes a pattern of gene expression contributing to enhanced invasion and suggests a role for chymase in mediating the biologic functions of miR-9.

Interestingly, miR-9 modulated the expression of other proteases in normal mast cells, including up-regulation of heparinase (HSPE). Heparinase is an endogylocosidase that functions in the degradation and release of heparan sulfate-bound growth factors [50]. Previous studies have shown that enzymatic cleavage of heparin sulfate by heparinase results in disassembly of the extracellular matrix and basement membrane dissolution, inducing structural modifications that loosen the extracellular matrix barrier and enable cell invasion [51]. Heparinase increases tumor invasion in both cell lines and spontaneous tumor models, through both extracellular matrix remodeling and increased peritumoral lymphangiogenesis [52]. Our data show that normal mast cells overexpressing miR-9 exhibit markedly increased HSPE expression, supporting the assertion that miR-9 may promote the metastatic phenotype by enhancing the proteolytic activity of a number of proteases important in physical remodeling of the extracellular matrix and activate mediators responsible for cell dissemination.

The present study investigated alterations in gene transcript expression affected by miR-9; however, these changes were not demonstrated at the protein level. Gene expression does not directly correlate with changes at the protein level and miRNAs may suppress protein expression by post-transcriptional silencing mechanisms that are not reflected in transcriptional profiling analyses. Furthermore, inhibition of miR-9 in canine mast cell lines would provide further convincing evidence of its importance in mast cell invasion. As such, identifying proteins altered by miR-9 that promote cell invasion and validating these targets in canine cell lines/tumors represents an area of ongoing investigation.

## Conclusion

In summary, the work presented here is the first to demonstrate that unique miRNA expression profiles correlate with the biological behavior of canine MCTs. Furthermore, overexpression of miR-9 is associated with aggressive biologic behavior of canine MCTs, possibly through the promotion of a metastatic phenotype as demonstrated by enhanced invasive behavior of normal and malignant mast cells and alteration of gene expression profiles associated with cellular invasion in the presence of enforced miR-9 expression. Future work to dissect the exact mechanisms through which miR-9 exerts the invasive phenotype is ongoing with the ultimate goal of identifying potential druggable targets for therapeutic intervention.

## Competing interest

The authors declare no competing financial interests.

## Authors’ contributions

Contribution: JF designed and performed research, analyzed data, and wrote manuscript; MDB and BKH assisted with mBMMC and primary MCT sample preparation; TYL generated preliminary data that led to work with miRNA and mast cells, assisted with cBMMC and primary MCT sample preparation; SV performed biostatistic analysis; WCK and CAL assisted in research design, oversaw data analysis, writing and editing of paper. All authors read and approved the final manuscript

## Pre-publication history

The pre-publication history for this paper can be accessed here:

http://www.biomedcentral.com/1471-2407/14/84/prepub

## Supplementary Material

Additional file 1 Clinical patient data. Click here for file
