# Supplementary material for: Overexpression of miR-9 in mast cells is associated with invasive behavior and spontaneous metastasis
Source: BMC Cancer. 2014 Feb 11;14:84. doi: 10.1186/1471-2407-14-84 (PMC3933481; doi:10.1186/1471-2407-14-84)
Supplement: Supplementary file 1 [file 1471-2407-14-84-S1.doc]

Sample	Breed	Sex	Primary Tumor Location	Local Recurrence and/or Site of Metastasis	Grade	Mitotic Index	Outcome
L1	Akita mix	FS	Neck	None	2	1	No recurrence, cause of death nasal tumor
L2	Golden Retriever	MC	Left tail base	None	2	1	No recurrence, cause of death osteosarcoma
L3	Labrador Retriever	MC	Right stifle	None	2	0	No recurrence
L4	Labrador Retriever	MC	Left dosal thorax	None	2	0	No recurrence
L5	Mixed breed	FS	Right caudal thorax	None	1	0	No recurrence
L6	Boxer	FS	R flank, L pinna, R lat stifle	None	2	0	No recurrence
L8	Boxer	FS	Right axilla	None	2	0	No recurrence
L7	Labrador Retriever	FS	Right ventral abdomen	None	2	0	No recurrence
L9	Golden Retriever	MC	Left flank	None	2	0	No recurrence
L10	Labrador Retriever	MC	Left caudal stifle	None	1	0	No recurrence, cause of death multifocal undifferentiated mesenchymal tumor
L11	Mixed breed	MC	Right palmar metacarpus	None	2	1	No recurrence
L12	Mixed breed	FS	L palmar metatarsus, L stifle	Popliteal LN	2	0	No recurrence or progressive metastatic disease, cause of death hemangiosarcoma
H1	Pit Bull Terrier	MC	Left medial thigh	Sublumbar LN	3	1-2	Died of Disease
H2	Akita mix	MC	Right maxillary lip	Mandibular LN, Retrobulbar Met	3	4	Died of Disease
H3	Doberman Pinscher	FS	Left stifle	Left inguinal LN	3	3-4	Died of Disease
H4	Mini Schnauzer	FS	Left shoulder	Local recurrence	3	20	Died of Disease
H5	Labrador Retriever	MC	Lateral thorax	Local recurrence, Spleen	2	1	Died of Disease
H6	Labrador Retriever	MC	Multifocal cutaneous	Regional LN, Spleen	3	>5	Died of Disease
H7	English Bulldog	MC	Right front interdigital	Prescapular LN	2	10	Died of Disease
H8	Beagle	FS	Multifocal cutaneous	Sublumbar/hypogastric LN, Spleen	3	NR	Died of Disease
H9	Mixed breed	FS	Cranial sternum	Local recurrence, Prescapular LN	2	8	Died of Disease
H10	Golden Retriever	MC	Multifocal cutaneous	De novo, multifocal cutaneous MCT, Popliteal LN	3	6-17	Died of Disease
H11	Shar pei	FS	L axilla, flank	Local recurrence, de novo cutaneous MCTs	3	<5	Died of Disease
H12	Shar pei	MC	R ear base	Prescapular LN	3	NR	Died of Disease
